# Supplementary material for: Effect of Prolong-life-with-nine-turn-method (Yan Nian Jiu Zhuan) Qigong on fatigue and gastrointestinal function in patients with chronic fatigue syndrome: Study protocol for a randomized controlled trial
Source: PLoS One. 2023 Nov 3;18(11):e0287287. doi: 10.1371/journal.pone.0287287 (PMC10624268; doi:10.1371/journal.pone.0287287)
Supplement: S2 File — (DOCX) [file pone.0287287.s002.docx]

**延年九转法干预慢性疲劳综合征的脑肠轴特征研究研究方案**

1. **研究背景**

慢性疲劳综合征（Chronic fatigue syndrome, CFS）是一种病因未明的、多系统病变疾病，其症状包括超过6个月无法解释的疲劳、活动后不适、神经功能障碍、免疫功能受损、胃肠道紊乱和泌尿生殖系统损伤^[1]^。据报道，欧洲CFS的患病率在0.1％至2.2％之间，中国CFS的总患病率则高达12.54%，并且由于初步诊断的不准确以及客观标志物的缺乏，一些CFS患者存在误诊、漏诊，CFS的患病率与发病率存在低估的风险^[2,3]^。CFS的多系统病变以及发病率的逐渐上升给个人、家庭乃至社会带来承重的经济负担，也引起了医学、心理学界的广泛关注^[4]^。

近年来研究表明消化性溃疡患者、炎症性肠病患者发生CFS的风险增加，其独立于性别、年龄以及合并症等提高CFS发病率的因素，并且CFS患者常伴发肠易激综合征^[5-7]^。本课题组在前期CFS相关研究中发现伴有胃肠症状的患者较其他CFS患者存在更加严重的生理、心理疲劳，这与CFS与胃肠道功能病的相关交叉研究的观点一致^[7]^。

随着脑-肠轴提出，CFS的发病机制进一步被明确，而调整胃肠功能的成为治疗CFS的新方向。脑功能变化是CFS与正常人区别的显著特征之一，CFS患者记忆（左海马旁回），运动（双侧苍白球），情绪（ACC）和更高级别的区域排序神经认知（ACC，AG和SFG）功能存在改变^[8]^。值得注意的是已有研究通过治疗抑郁症伴有和不伴有胃肠道症状的患者发现海马旁回的改变与胃肠道症状关系密切，但与抑郁症状无关^[9]^。这些脑区功能的相关变化与脑肠轴的异常调控密切相关。据报道CFS患者肠道菌群失调，在门水平上，厚壁菌门、放线菌门相对丰度降低，拟杆菌门、变形菌门相对丰度提高；在属水平上拟杆菌属、梭菌属、Pseudoflavonifractor属、Phascolarctobacterium属等菌属相对丰度提高，粪杆菌属、瘤胃球菌属、Roseburia属、Coprococcus属、Bacteroides vulgatus属、双歧杆菌属等菌属相对丰度降低，是CFS患者胃肠症状高发的内在病理因素^[7]^。事实上，Pseudoflavonifractor属相对丰度增加、Coprococcus属相对丰度的降低与CFS患者情绪低落、动机减少有关，粪杆菌属、瘤胃球菌属、Roseburia属相对丰度的下降可能与CFS合并肠易激综合征关系密切，未分类的拟杆菌属相对丰度增加可能是没有合并症的CFS特异性标志物。提示肠道菌群对于脑功能存在重要的影响，脑-肠轴在其中发挥着重要作用^[10]^。5-羟色胺（5-HT）、神经肽Y(Neuropeptide Y, NPY)、P 物质(Substance P, SP)、降钙素基因相关肽(Calcitonin Gene-Related Peptide, CGRP)等脑肠肽作为广泛分布于大脑及胃肠道的肽类物质，是沟通胃肠道及大脑的重要物质，也是脑肠轴的重要组成部分，其血液浓度的改变是CFS患者脑肠轴异常的客观证据^[11,12]^。

中医认为脾胃是CFS主要病位。在古代中医医籍中常用“懈怠”、“懈情”、“四肢沉重”、“四肢劳倦”、“四肢瘫软”、“四肢不用”等描述CFS相关症状，而这些词汇也多于与脾胃相关^[13]^。《素问·本病论》记载：“人饮食、劳倦即伤脾”；《素问·脏气法时论》“脾病者，身重善肌肉痿，足不收行，善瘈，脚下痛”；《素问·本神》指出：“怵惕思虑者则伤神，神伤则恐惧流淫而不止…脾，愁忧而不解则伤意，意伤则悗乱，四肢不举…”；《素问·玉机真藏论》言：“五脏者，皆禀气于胃，胃者五脏之本也”。临床上常见心脾两虚证、肝郁脾虚证、脾胃气虚证、气血两虚证等证^[14]^。脾胃损伤可促进CFS的发生，而CFS又可进一步损伤脾胃，这种相互的影响不仅造成CFS的迁延难愈，也是造成多系统病变的重要原因，因此中医常从脾胃论治CFS。

然而，从胃肠道入手治疗CFS的非药物疗法仍处于探索阶段。国外相关非药物疗法研究更关注CFS的疼痛、睡眠，冥想、运动等治疗手段被广泛用于CFS治疗，但认知行为疗法作为肠易激综合征治疗的重要手段，也被认为是CFS公认的治疗方法，为存在胃肠道症状的患者提供了有效的治疗手段^[15]^。在中医非药物疗法中针刺、艾灸从脾论治的临床研究已经表明对CFS患者的疲劳、焦虑、抑郁程度、睡眠质量改善效果良好^[16]^，在机制研究中也发现采用腹部推拿可以影响CFS模型大鼠脑内相关因子、蛋白的表达，由此改善大鼠的行为学特征^[16]^。但很少有研究从脾入手探索推拿手法、功法的临床疗效。传统功法作为中医非药物疗法在防治CFS已经初见成效，其身心同调的独特优势是改善CFS患者疲劳、失眠、抑郁的有效方法，进一步提供高质量的临床试验以及机制的深入挖掘有利于CFS患者更好的恢复日常生活^[16]^。延年九转法是一种腹部按摩结合导引及静功的传统功法。由于该功法九式中八式以按摩为主，常用转圈式进行自我按摩，且其功法开创者方开寿近百岁，故称其功法为“延年九转法”。清代韩德元跋认为该功法具有治疗失眠、“虚劳及停饮者”的作用，受术者“无不愈”。本课题组通过延年九转法干预CFS的研究中也表明延年九转法对CFS患者精神疲劳、生理疲劳程度、CFS患者的兴趣以及活动具有潜在益处^[20]^，但延年九转法能否有效改善CFS胃肠道功能，并由此调整脑-肠轴治疗CFS仍需进一步研究。

为明确延年九转法对CFS的治疗作用，本研究将针对伴有胃肠症状的CFS患者。通过观察延年九转法和认知行为疗法对伴胃肠症状的CFS患者疲劳程度、生活质量、胃肠道症状的影响，并检测肠道菌群多样性、脑功能以及脑肠肽指标，探索延年九转法干预CFS的脑-肠轴效应，为防治CFS提供临床证据。

课题组多年来一直从事CFS的非药物疗法防治工作，取得较好的临床效果。已完成国家自然科学基金、国家体育总局、上海市科委、上海市教委等多项课题，取得预期成果，并在核心期刊发表多篇相关研究论文，具有一定的科研能力和较扎实的前期工作基础。课题负责人主要从事中医推拿、功法防治CFS的研究，近10年一直从事推拿防治失眠及慢性疲劳综合征的相关研究工作。本研究承接既往研究，提供高质量临床证据，揭示功法作用机制。

**参考文献**

[1] B-M Carruthers, van de Sande M-I, De Meirleir K-L, et al. Myalgic encephalomyelitis: International Consensus Criteria[J]. J Intern Med, 2011, 270(4): 327-338.

[2] F Estévez-López, Mudie K, Wang-Steverding X, et al. Systematic Review of the Epidemiological Burden of Myalgic Encephalomyelitis/Chronic Fatigue Syndrome Across Europe: Current Evidence and EUROMENE Research Recommendations for Epidemiology[J]. J Clin Med, 2020, 9(5).

[3] 伍侨，高静，柏丁兮，等. 中国人群慢性疲劳综合征患病率的Meta分析[J]. 右江医学, 2020, 48(10): 727-735.

[4] 孙增坤，蒙玲莲，何裕民. 从“重启慢性疲劳综合征研究”受关注谈起[J]. 科学通报, 2019, 64(23): 2379-2385.

[5] Kuo ChienFeng, Leiyu Shi, ChengLi Lin, et al. How peptic ulcer disease could potentially lead to the lifelong, debilitating effects of chronic fatigue syndrome: an insight.[J]. Scientific reports, 2021, 11(1).

[6] Shin-Yi Tsai, Chen Hsuan-Ju, Lio Chon-Fu, et al. Increased risk of chronic fatigue syndrome in patients with inflammatory bowel disease: a population-based retrospective cohort study[J]. BioMed Central, 2019, 17(1).

[7] Dorottya Nagy-Szakal, Williams Brent-L, Mishra Nischay, et al. Fecal metagenomic profiles in subgroups of patients with myalgic encephalomyelitis/chronic fatigue syndrome[J]. BioMed Central, 2017, 5(1).

[8] J Boissoneault, Letzen J, Lai S, et al. Abnormal resting state functional connectivity in patients with chronic fatigue syndrome: an arterial spin-labeling fMRI study[J]. Magn Reson Imaging, 2016, 34(4): 603-608.

[9] P Liu, Li G, Zhang A, et al. The prognosis and changes of regional brain gray matter volume in MDD with gastrointestinal symptoms[J]. Neuropsychiatr Dis Treat, 2019, 151181-1191.

[10] 王建楠，曾艳平. 肠道菌群在慢性疲劳综合征中的作用研究进展[J]. 中国微生态学杂志, 2021, 33(04): 478-481.

[11] 詹婷瑜. 针灸结合耐力性运动治疗慢性疲劳综合征的临床观察及对血清5-HT的影响[D]. 福建中医药大学, 2021.

[12] 陈辉，蔡憐环，何伟秀. 基于脑肠轴学说的太极云手对慢性疲劳综合征患者胃肠功能的影响[J]. 中国当代医药, 2021, 28(30): 143-146.

[13] 李外常章富高云艳. 中医对慢性疲劳综合征的认识与治疗[J]. 中国中医基础医学杂志, 2004, (02): 63-65.

[14] 彭敏，马宏博，司国民. 慢性疲劳综合征中医证候及证素特点的文献评析[J]. 中国中西医结合杂志, 2014, 34(06): 691-693.

[15] Committee-on-the-Diagnostic Fatigue, Syndrome, Populations Board-on-the-Health, et al. Beyond Myalgic Encephalomyelitis/Chronic Fatigue Syndrome: Redefining an Illness[M]. Washington (DC): National Academies Press (US), 2015.

[16] 张春燕，赵淑华，李利，等. 近五年中医外治法治疗慢性疲劳综合征研究进展[J]. 亚太传统医药, 2019, 15(12): 203-205.

[17] 李华南，韩一豪，刘洋，等. 腹部推拿对慢性疲劳综合征模型大鼠海马区Ca~(2+)浓度及MAPK、ERK蛋白表达的影响[J]. 辽宁中医杂志, 2019, 46(09): 1817-1820.

[18] 李华南，韩一豪，刘洋，等. 腹部推拿对慢性疲劳综合征模型大鼠行为学及海马区BDNF、CREB mRNA表达的影响[J]. 中国中医基础医学杂志, 2019, 25(06): 750-753.

[19] 谢芳芳，管翀，成子己，等. 传统功法干预慢性疲劳综合征的研究进展[J]. 中医药导报, 2020, 26(11): 98-100.

[20] 谢芳芳，王伟健，管翀，等. 延年九转法对慢性疲劳综合征患者疲劳和生活质量影响的临床研究[J]. 时珍国医国药, 2020, 31(12): 2951-2955.

[21] 吴位东. 精神分裂症患者脑区功能与肠道菌群改变的关联性研究[D]. 内蒙古医科大学, 2019.

[22] J-B Prins, Bleijenberg G, Bazelmans E, et al. Cognitive behaviour therapy for chronic fatigue syndrome: a multicentre randomised controlled trial[J]. Lancet, 2001, 357(9259): 841-847.

[23] 金玉莲，黄海晓，赵娜，等. 认知行为疗法在慢性疲劳综合征康复中的应用效果[J]. 浙江医学, 2017, 39(12): 1036-1038.

[24] A Deale, Chalder T, Marks I, et al. Cognitive behavior therapy for chronic fatigue syndrome: a randomized controlled trial[J]. Am J Psychiatry, 1997, 154(3): 408-414.

[25] A Janse, Worm-Smeitink M, Bleijenberg G, et al. Efficacy of web-based cognitive-behavioural therapy for chronic fatigue syndrome: randomised controlled trial[J]. Br J Psychiatry, 2018, 212(2): 112-118.

1. **研究目的**

通过随机对照研究，观察延年九转法对伴有胃肠症状的慢性疲劳综合征患者的治疗效果，并通过fMRI、16S rRNA技术探索延年九转法治疗CFS的脑-肠轴机制。

1. **研究方法**

随机、对照临床研究。

1. **研究对象**
2. **病例来源**

在岳阳医院粘贴海报、微信公众号推广及社区CFS宣讲招募以疲劳为主诉并伴有腹痛、腹胀、恶心、早饱、呕吐、腹泻、排便困难、消化不良其中1项或以上1项以上症状至少3个月的受试者。

1. **样本量计算的依据**

本研究中，样本量的计算基于疲劳量表分数的改善。假设：

H_0_：μ_1_=μ_2_

H_1_：μ_1_≠μ_2_

μ_1_为延年九转组疲劳量表平均得分，μ_2_为认知行为组疲劳量表平均得分

根据前期研究^[20]^，计算对照组疲劳量表平均得分较基线变化，两组疲劳量表得分最终变化差异为2.216，标准差为3.172.使用均值比较法得出：

n=$2\times{[\frac{（Z_{\alpha/2}＋Z_{\beta})\times}{}]}^{2}$

=$2\times{[\frac{（1.96＋1.282)\times3.172}{2.216}]}^{2}$

=43.07≈43

（α=0.05、β=0.9，双边检测）

延年九转组、认知行为组分配相同数量的患者，每组43例。考虑10%的人员流失，每组需要48名受试者。

为探索CFS脑-肠轴特征，根据受试者意愿选取每组选取15名受试者进行fMRI及肠道菌群检查，进行脑-肠轴机制探索。根据既往相关研究显示每组12至15名患者在fMRI研究以及微生物研究中已经具有统计效力^[21]^。

1. **诊断标准**

3.1 CFS诊断标准

根据1994年美国疾病控制与预防中心制定的Fukuda诊断标准作为CFS的诊断标准。

3.1.1主要症状：疲劳，经临床评价后无法解释的病史不少于6个月的严重慢性疲劳，疲劳经休息后不能得到缓解。

3.1.2伴随症状：同时至少具备下列八项中的四项

(1)记忆力或注意力下降，其严重性导致职业能力、接受教育能力、社会活动能力及个人生活能力等方面较患病前有实质性下降。

(2)咽痛

(3)颈部或腋窝淋巴结触痛

(4)肌肉疼痛

(5)不伴有红肿多关节疼痛

(6)发作方式、类型及严重程度与以前不同的头痛

(7)睡眠后不能回复精力

(8)劳累后肌痛超过24小时

1. **纳入标准**

4.1符合慢性疲劳综合征诊断标准

4.2出现腹痛、腹胀、恶心、早饱、呕吐、腹泻、排便困难、消化不良其中1项或以上1项以上症状至少3个月

4.3年龄≥20岁且≤60岁，性别不限，右利手。

4.4理解、同意参加本研究并签署知情同意

1. **排除标准**

5.1严重的心脑血管疾病，内分泌系统疾病，运动系统疾病，自身免疫性疾病，传染病，糖尿病或其他精神疾病。

5.2患有肿瘤、结核、风湿或类风湿性关节炎、痛风、关节创伤，或有脑外伤伴意识丧失的病史。

5.3明确诊断胃肠道器质性疾病，肝肾功能不全的受试者。

5.4近4周饮食出现大幅变化

5.5除阑尾炎手术以外做过其他手术的受试者

5.6研究前3-6个月，服用过抗生素、类固醇激素、中草药

5.7有闭锁恐惧症、心脏起搏器、除颤器、心脏支架、宫内节育器等MRI禁忌症者。

5.8孕妇或哺乳期妇女，药物成瘾，重金属中毒或类似状况。

1. **剔除、中止和脱落标准**

6.1误排、误纳；

6.2受试者依从性差，未按规定进行治疗；

6.3病历资料不全，影响疗效评价；

6.4受试者自行退出；

6.5受试者试验过程中发生不良事件或严重不良事件，不适宜继续参加研究；

6.6受试者疾病严重恶化或发生了某些合并症、并发症和特殊生理变化，不适合继续参加本研究；

6.7研究者认为不适合继续参加本研究者。

1. **试验分组**

将96例CFS患者按照1:1的比例随机分为试验组和对照组。

1. **治疗方案**

**1.对照组：认知行为组**

1.1方式：认知行为疗法

1.2周期：12周

1.3频次：每周线下集中心理辅导1次，每次1小时，并布置家庭作业。

1.4内容：

CFS的认知行为疗法是基于CFS中疲劳持续性因素的统计测试模型^[21]^。

认知行为治疗是协作性的、教育性的、协商性的，并且强调行为。根据前期研究，第1周至第3周为认知探查，涉及让患者参与治疗并提供详细的治疗原理。评估提出的问题，并记录受试者每小时的活动、休息和疲劳细节。同时，鼓励受试者日记记录（每天记录事件、感觉、疲劳和肌肉紧张）。第4周至第7周为运用三栏表、垂直箭头法等技术，寻找核心信念。在整个过程中让患者主动找出自己的不良认知与错误信念，咨询师只进行分析、例举说明、角色扮演、引导启发等而不做对错判断。第8周至第12周实施认知重建。通过渐进式暴露疗法、分级活动计划，认知疲劳严重程度、认知疲劳的环境特异性、认知疲劳所致的影响与结果、认知休息与运动解压、饮食调整对疲劳的缓解作用^[23-25]^。

**2.****试验组：延年九转法**

2.1方式：延年九转法

2.2周期：12周

2.3频次：每周线下集中带教1次，每次1小时，其余6天患者按照标准视频自行操作，每次30分钟。

2.4内容：

参照全国普通高等中医药类精编教材《推拿功法学》

第一式：以两手中三指按心窝，由左顺摩圆转21次。

第二式：以两手中三指由心窝顺摩而下，且摩且走，摩至脐下高骨为度。

第三式：以两手中三指由高骨处向两边分摩而上，且摩且走，摩至心窝。两手交接为度。

第四式：以两手中三指由心窝由下，直推至高骨，共21次。

第五式：以右手由左绕摩脐腹21次。

第六式：以左手由右绕摩脐腹21次。

第七式：左手置左边软肋下腰肾处，大指向前，四指托后，轻轻捏定；右手中三指自左乳下直推至腹股沟21次。

第八式：右手置右边软肋下腰肾处，大指向前，四指托后，轻轻捏定；左手中三指自右乳下直推至腹股沟21次。

第九式：将一至八式依次作完为1度，每次应连作7度。推毕盘坐，以两手握固分按两膝上。脚趾微屈。将上身顺时针摇转21次。然后，逆时针摇转21次。

**3. 质量控制**

试验组：患者入组后，由医生作为期3天的延年九转法培训和刺激量标定，刺激量均值(0.5±0.1)kg。制作标准视频及操作记录本，发放给每位受试者并要求受试者及时填写操作记录本并线上传给质量控制负责人。受试者将使用微信每周进行拍照，研究人员由此了解参与者锻炼情况。对照组：患者入组后，由心理咨询师面对面进行心理评估并建立治疗联盟。所有的认知行为治疗将由1名心理咨询师及科研助手辅助完成，治疗访谈内容由科研助手负责记录并递交质量控制负责人。受试者将被要求根据治疗内容记录日常锻炼或学习信息。

1. **观察指标**
2. **主要指标**

多维疲劳评分量表（MFI-20）：MFI-20将用于评估疲劳程度，包括20个项目，涉及五个维度：正直、生理、精神、活动和热情。每项采用Likert5评分法，完全符合1分，完全不符合5分。其中10个描述疲劳的项目得分为正，10个描述非疲劳的项目得分为反，总分为20-100分。分数越高，疲劳程度越重。

1. **次要指标**

2.1胃肠道症状评定量表（GSRS）：GSRS包括15个项目，涉及五个症状群：反流、腹痛、消化不良、腹泻和便秘。每项采用Likert 7级评分法，完全符合1分，完全不符合7分。

2.2 健康调查量表36项（SF-36）：共有36个项目和9个维度，包括：生理功能、身体疼痛、一般健康、活力、社会功能、情绪功能、心理健康和健康变化。九个维度中的每一项都单独编码和求和，并用0-100表示。分数越高，可能的健康状况越好。

2.3营养KAP（知识、态度、行为）问卷：问卷更据前期研究制定。由三大部分组成，分别是营养知识、营养态度、营养行为，每个部分单独编码求和，三大部分总分为100，分数越高代表参与人可能的营养状况越好。

2.4食物频率问卷：分为两部分，食物的种类和各种食物在一定时间内的摄入频率。通过问卷回答结果计算获得过去一段时间内个体的食物摄入量的数据。了解调查对象的膳食模式以及饮食习惯。

2.5Bristol大便分型量表（BSFS）：BSFS用于判断粪便的形状。将大便分为七种大便类型之一，从1型（硬块）到7型（水样腹泻）。

2.6脑肠肽检测：血液将在岳阳中西医结合医院采集。凝固后，在3000 rpm下离心5分钟后取血清，并储存在-80℃的冰箱中。采用酶联免疫吸附试验（ELISA）检测血清5-HT、NPY、SP、CGRP水平。

2.7静息态功能磁共振检测：功能磁共振数据采集将使用岳阳中西医结合医院的3.0T超导磁共振Magnetom skyra成像系统，该系统具有32个头部专用线圈。粗体功能磁共振成像扫描参数如下：全脑，重复时间（TR） = 2000 毫秒，回音时间（TE） = 30 ms，视野（FOV） = 250mm× 250mm，翻转角度（FA） = 90°，基体=256mm×256mm，切片厚度/间隙 = 4.0/1 mm，体素大小=1.0  × 1.0  × 1.0 ，轴向切片 = 33层，总共240层。在功能磁共振成像数据收集之前，参与者将被要求休息10分钟。在数据采集过程中，参与者将仰卧并闭上眼睛。泡沫头用于减少头部移动，耳塞用于减少噪音影响，参与者应保持清醒，避免思考。两组参与者的功能磁共振成像数据将在基线检查时和治疗12周后进行测量。

2.8肠道菌群检测：从医院参与者身上采集新鲜粪便样本，并在-80℃冰箱中冷冻3小时。E.Z.N.A.土壤DNA试剂盒（Omega Bio-Tek，美国佐治亚州诺克罗斯）将用于微生物DNA提取。16S rRNA基因的V3-V4高变区将通过PCR使用338F（5'-ACTCCTACGGGGAGGGCAGAG-3'）和806R（5'-GGACTACHVGGGTWTCTAAT-3'）进行扩增，然后使用Illumina MiSeq PE300测序。将原始数据上传至NCBI SRA数据库。

1. **疗效评价标准**

疗效评价标准(采用MFI-20减分率进行判定）

治愈：临床主症及兼症完全消失，MFI-20评分减少≥95%。

显效：临床主症及兼症消失≥2/3，MFI-20评分减少≥70%。

有效：临床主症及兼症消失≥1/3，MFI-20评分减少≥30%。

无效：临床主症及兼症消失＜1/3，MFI-20评分减少＜30%。

有效率=[（治疗前评分-治疗后评分）/治疗前评分]×100%

总有效率=治愈率+显效率+有效率

1. **统计分析**

将所有数据输入Microsoft Excel，建立数据库。并采用SPSS24.0 统计软件进行数据分析。对正态分布的计量资料采用“均数±标准差”表示，不符合正态分布的计量资料采用“中位数、四分位间距”表示，对计数资料采用“频数，百分比”表示；若满足正态分布和方差齐性检验，两组研究前、干预后6周、干预12周、随访6周，多个时间点变化的比较采用重复测量设计资料的方差分析，若不满足正态分布和方差齐性，采用H检验。检验标准α为0.05，当P＜0.05为差异有统计学意义。
