# Supplementary material for: Effect of Prolong-life-with-nine-turn-method (Yan Nian Jiu Zhuan) Qigong on fatigue and gastrointestinal function in patients with chronic fatigue syndrome: Study protocol for a randomized controlled trial
Source: PLoS One. 2023 Nov 3;18(11):e0287287. doi: 10.1371/journal.pone.0287287 (PMC10624268; doi:10.1371/journal.pone.0287287)
Supplement: S3 File — (DOCX) [file pone.0287287.s003.docx]

**Research Scheme of the Brain gut Axis Characteristics of the Chronic Fatigue Syndrome Intervened by Yannian Jiuzhuan Method**

**Research background**

Chronic fatigue syndrome (CFS) is a disease with unknown etiology and multiple system diseases. Its symptoms include unexplained fatigue, post activity discomfort, neurological dysfunction, impaired immune function, gastrointestinal tract disorder and urinary and reproductive system damage for more than 6 months . It is reported that the prevalence of CFS in Europe is between 0.1% and 2.2%, while the total prevalence of CFS in China is as high as 12.54%. Moreover, due to the inaccuracy of the initial diagnosis and the lack of objective markers, some CFS patients are misdiagnosed and missed diagnosis, and the prevalence and incidence rate of CFS are at risk of underestimation. The multiple system lesions of CFS and the gradual rise of its incidence rate have brought heavy economic burden to individuals, families and even society, and have also attracted extensive attention from the medical and psychological circles [4].

In recent years, studies have shown that patients with peptic ulcer and inflammatory bowel disease have an increased risk of CFS, which is independent of gender, age, comorbidities and other factors that increase the incidence rate of CFS, and CFS patients are often accompanied by irritable bowel syndrome . In the previous CFS related research, our research team found that patients with gastrointestinal symptoms had more serious physiological and psychological fatigue than other CFS patients, which was consistent with the view of cross study on CFS and gastrointestinal functional diseases .

With the introduction of brain gut axis, the pathogenesis of CFS has been further clarified, and the adjustment of gastrointestinal function has become a new direction of treatment for CFS. The change of brain function is one of the significant features that distinguish CFS from normal people. The function of memory (left parahippocampal gyrus), movement (bilateral globus pallidus), emotion (ACC) and higher level regional sorting neurocognition (ACC, AG and SFG) in CFS patients has changed. It is worth noting that some studies have found that the changes of parahippocampal gyrus are closely related to gastrointestinal symptoms, but not related to depressive symptoms through the treatment of patients with and without gastrointestinal symptoms of depression. The functional changes of these brain regions are closely related to the abnormal regulation of brain gut axis. It is reported that the intestinal flora of CFS patients is maladjusted. At the phyla level, the relative abundance of Firmicutes and Actinomycetes decreases, while the relative abundance of Bacteroides and Proteus increases; At the genus level, the relative abundance of Bacteroides, Clostridium, Pseudolava, Phascolarctobacter and other genera is increased, and the relative abundance of Fecal Bacillus, Rumen Coccus, Roseburia, Coprococcus, Bacteroides vulgatus and Bifidobacterium is reduced, which is the internal pathological factor for the high incidence of gastrointestinal symptoms in CFS patients. In fact, the increase of the relative abundance of Pseudo lavanicator and the decrease of the relative abundance of Coprococcus are related to the depression and motivation of CFS patients. The decrease of the relative abundance of Fecal Bacillus, Ruminococcus and Roseburia may be closely related to CFS with irritable bowel syndrome. The increase of the relative abundance of unclassified Bacteroides may be a specific marker of CFS without complications. It suggests that intestinal flora has an important influence on brain function, in which brain gut axis plays an important role [10]. As peptides widely distributed in brain and gastrointestinal tract, 5-HT, neuropeptide Y, substance P, and calcitonin gene related peptide (CGRP) are important substances to communicate gastrointestinal tract and brain, and also important components of brain gut axis. Changes in blood concentration are objective evidence of abnormal brain gut axis in CFS patients .

TCM believes that spleen and stomach are the main disease location of CFS. In ancient Chinese medical books, CFS related symptoms are often described by "slackness", "slackness of feeling", "heaviness of limbs", "fatigue of limbs", "paralysis of limbs", "disuse of limbs", and these words are more related to the spleen and stomach . Suwen · Theory of this Disease records that "people's diet and fatigue hurt the spleen"; "Su Wen · Theory of Zang Qi Method": "People with spleen diseases are good at muscle flaccidity, and their feet do not stop. They are good at walking, and their feet are painful"; Su Wen · Ben Shen points out: "Those who are afraid of thinking will hurt their minds, while those who are scared will not stop worrying... The spleen will be hurt if they are worried but not understood, and the mind will be confused if they are hurt, and their limbs will not be lifted..."; "Su Wen · Jade Mechanism Theory of True Collection" said: "The five internal organs are all endowed with Qi in the stomach, and the stomach is the origin of the five internal organs". Clinically, there are many syndromes such as deficiency of both heart and spleen, liver depression and spleen deficiency, spleen stomach qi deficiency, and qi blood deficiency [14]. Spleen and stomach injury can promote the occurrence of CFS, and CFS can further damage the spleen and stomach. This mutual influence not only causes the persistence of CFS, but also an important reason for multi system diseases. Therefore, traditional Chinese medicine often treats CFS from the spleen and stomach.

However, the non drug therapy for CFS from gastrointestinal tract is still in the exploration stage. Relevant foreign non drug therapy studies pay more attention to the pain, sleep, meditation, exercise and other treatment methods of CFS, which are widely used in CFS treatment. However, cognitive behavioral therapy, as an important means of treatment of irritable bowel syndrome, is also considered as a recognized treatment method of CFS, providing effective treatment methods for patients with gastrointestinal symptoms . The clinical study of acupuncture and moxibustion treating CFS patients from the spleen in the non drug therapy of traditional Chinese medicine has shown that it has a good effect on improving the fatigue, anxiety, depression and sleep quality of CFS patients. In the mechanism study, it is also found that abdominal massage can affect the expression of related factors and proteins in the brain of CFS model rats, thereby improving the behavioral characteristics of rats. However, few studies have explored the clinical efficacy of massage techniques and exercises from the spleen. As a non drug therapy of traditional Chinese medicine in the prevention and treatment of CFS, the unique advantage of the physical and mental coherence is to improve the fatigue, insomnia and depression of CFS patients, and further provide high-quality clinical trials and in-depth exploration of mechanisms, which is conducive to better recovery of daily life of CFS patients. The Nine Turn Method is a traditional method of abdominal massage combined with guidance and static exercise. As the eight of the nine movements of this skill are mainly massage, and the self massage is often performed in a circle, and Fang Kaishou, the founder of this skill, is nearly 100 years old, so it is called the "Nine Turn Method for Prolonging Years". Han Deyuan's postscript in the Qing Dynasty believed that this skill had the effect of treating insomnia, "empty fatigue and those who stopped drinking", and that the recipient of the skill "would recover". Our research group's research on the intervention of CFS through the method of prolonging the years and turning nine times also shows that the method of prolonging the years and turning nine times has potential benefits for the mental fatigue and physiological fatigue degree of CFS patients, the interest and activities of CFS patients, but whether the method of prolonging the years and turning nine times can effectively improve the gastrointestinal function of CFS, and thus adjust the brain gut axis to treat CFS still needs further research.

In order to clarify the therapeutic effect of Jiuzhuan Method on CFS, this study will focus on CFS patients with gastrointestinal symptoms. To explore the brain gut axis effect of Yannian Jiuzhuan Method on CFS by observing the effects of Yannian Jiuzhuan Method and cognitive behavioral therapy on fatigue, quality of life, gastrointestinal symptoms of CFS patients with gastrointestinal symptoms, and detecting intestinal flora diversity, brain function, and brain gut peptide indicators, so as to provide clinical evidence for the prevention and treatment of CFS.

The research group has been engaged in the prevention and treatment of non drug therapy of CFS for many years, and has achieved good clinical results. It has completed a number of projects such as the National Natural Science Foundation of China, the General Administration of Sport of China, the Shanghai Municipal Science and Technology Commission, and the Shanghai Municipal Education Commission, and has achieved the expected results. It has published a number of related research papers in core journals, and has a certain scientific research ability and a solid foundation for early work. The person in charge of the project is mainly engaged in the research on the prevention and treatment of CFS by traditional Chinese massage and exercises, and has been engaged in the research on the prevention and treatment of insomnia and chronic fatigue syndrome by massage for the past 10 years. This study undertakes previous research, provides high-quality clinical evidence, and reveals the mechanism of action of qigong.

**research objective**

To observe the therapeutic effect of Yannian Jiuzhuan Method on chronic fatigue syndrome patients with gastrointestinal symptoms through a randomized controlled study, and to explore the brain gut axis mechanism of Yannian Jiuzhuan Method in treating CFS through fMRI and 16S rDNA technology.

**research method**

Randomized and controlled clinical study.

**Research object**

**Source of cases**

Paste posters in Yueyang Hospital, promote wechat official account and CFS in the community to recruit subjects with fatigue as the main complaint and accompanied by abdominal pain, abdominal distension, nausea, early satiety, vomiting, diarrhea, defecation difficulties and indigestion for at least 3 months.

Basis for sample size calculation

In this study, the calculation of sample size is based on the improvement of fatigue scale scores. Assumptions:

H0： μ 1= μ two

H1： μ 1≠ μ two

μ 1 is the average score of the fatigue scale of the nine turn group, μ 2 is the average score of cognitive behavior group fatigue scale

According to the previous study [20], the average score of the fatigue scale of the control group was calculated to change from the baseline. The final difference between the two groups was 2.216, and the standard deviation was 3.172. The mean comparison method was used to obtain:

n=$2\times{[\frac{（Z_{\alpha/2}＋Z_{\beta})\times}{}]}^{2}$

=$2\times{[\frac{（1.96＋1.282)\times3.172}{2.216}]}^{2}$

=43.07≈43

（ α= 0.05、 β= 0.9, bilateral detection)

The Jiu Zhuan group and cognitive behavior group were assigned the same number of patients, 43 patients in each group. Considering 10% staff turnover, 48 subjects are required for each group.

In order to explore the characteristics of the brain gut axis of CFS, 15 subjects in each group were selected according to the wishes of the subjects for fMRI and intestinal flora examination to explore the brain gut axis mechanism. According to previous relevant studies, 12 to 15 patients in each group have been statistically effective in fMRI research and microbial research [21].

**diagnostic criteria**

**CFS diagnostic criteria**

According to the Fukuda diagnostic criteria formulated by the US Centers for Disease Control and Prevention in 1994, it is used as the diagnostic criteria for CFS.

1 Main symptoms: fatigue, serious chronic fatigue with a history of no less than 6 months that cannot be explained after clinical evaluation, and fatigue cannot be relieved after rest.

2 Accompanying symptoms: at least four of the following eight

(1) The severity of the decline in memory or attention leads to a substantial decline in professional ability, educational ability, social activity ability and personal living ability compared with that before the illness.

(2) Pharyngodynia

(3) Neck or axillary lymph node tenderness

(4) Muscle pain

(5) Without redness, swelling and pain

(6) Headache with different attack mode, type and severity

(7) Unable to recover energy after sleep

(8) Muscle pain exceeds 24 hours after fatigue

**Inclusion criteria**

1 Meet the diagnostic criteria of chronic fatigue syndrome

2 At least 3 months after onset of abdominal pain, abdominal distension, nausea, early satiety, vomiting, diarrhea, difficulty in defecation and dyspepsia

3 Age ≥ 20 and ≤ 60, regardless of gender, right handed.

4 Understand and agree to participate in the study and sign the informed consent

**Exclusion criteria**

1 serious cardiovascular and cerebrovascular diseases, endocrine system diseases, motor system diseases, autoimmune diseases, infectious diseases, diabetes or other mental diseases.

2 Have a history of tumor, tuberculosis, rheumatism or rheumatoid arthritis, gout, joint trauma, or brain trauma with loss of consciousness.

3 Subjects with definite diagnosis of gastrointestinal organic diseases and liver and kidney dysfunction.

4 Significant changes in diet in recent 4 weeks

5 Subjects who have undergone surgery other than appendicitis surgery

6 Take antibiotics, steroid hormones and Chinese herbal medicine 3-6 months before the study

7 Those with MRI contraindications such as atresia phobia, cardiac pacemaker, defibrillator, cardiac stent, intrauterine device, etc.

8 Pregnant women or lactating women, drug addiction, heavy metal poisoning or similar conditions.

**Rejection, suspension and shedding criteria**

1 Incorrect discharge and acceptance;

2 The subject's compliance is poor, and the treatment is not carried out as required;

3 Incomplete medical records affect the efficacy evaluation;

4 The subject withdraws himself/herself;

5 Adverse events or serious adverse events occurred to the subject during the test, and it is not suitable to continue to participate in the study;

6 The subject's disease has seriously deteriorated or some complications, complications and special physiological changes have occurred, so it is not suitable to continue to participate in this study;

7 The researcher believes that it is not suitable to continue to participate in this study.

**Test grouping**

96 CFS patients were randomly divided into test group and control group according to 1:1 ratio.

**Treatment plan**

**Control group: cognitive behavior group**

1 Method: cognitive behavioral therapy

2 Period: 12 weeks

3 Frequency: offline centralized psychological counseling once a week, one hour each time, and homework assignments.

4 Content:

Cognitive behavioral therapy of CFS is based on the statistical test model of fatigue persistence factors in CFS.

Cognitive behavior therapy is cooperative, educational, consultative, and emphasizes behavior. According to the previous study, the first week to the third week are cognitive explorations, involving involving patients in treatment and providing detailed treatment principles. Evaluate the questions raised and record the details of the subject's activity, rest and fatigue every hour. At the same time, the subjects were encouraged to keep diaries (recording events, feelings, fatigue and muscle tension every day). From week 4 to week 7, three column table and vertical arrow method were used to find the core beliefs. In the whole process, let the patient actively find out their own bad cognition and false belief, and the consultant only conducts analysis, illustration, role play, guidance and inspiration, instead of making right or wrong judgments. Cognitive reconstruction was carried out from week 8 to week 12. Through progressive exposure therapy, graded activity plan, the severity of cognitive fatigue, the environmental specificity of cognitive fatigue, the effects and results caused by cognitive fatigue, cognitive rest and exercise decompression, and the relieving effect of diet adjustment on fatigue.

**Test group: Nine year extension method**

1 Method: Nine year extension method

2 Period: 12 weeks

3 Frequency: offline centralized teaching once a week, 1 hour each time, and the rest 6 days, 30 minutes each time, according to the standard video.

4 Content:

Refer to the refined textbook of the national general higher traditional Chinese medicine Science of Massage

The first movement: press the heart socket with two hands and three fingers, and turn 21 times from left to right.

The second move: rub the three fingers of two hands down from the heart socket, and then rub them to the high bone under the umbilicus.

The third move: Use two hands and three fingers to rub from the high bone to the two sides, and rub and walk to the heart nest. Hand to hand is degree.

The fourth move: push the three fingers of two hands from the heart socket down to the high bone for 21 times.

The fifth type: rub the navel abdomen with the right hand from the left for 21 times.

The sixth form: Rub the navel abdomen with the left hand from the right for 21 times.

The seventh move: place the left hand at the lower waist and kidney of the left soft rib, with the big finger forward and the four fingers behind, gently pinch it; The middle third finger of the right hand is pushed directly from the left breast to the groin for 21 times.

The eighth move: place the right hand at the lower waist and kidney of the right soft rib, with the big finger forward and the four fingers behind, gently pinch it; The middle third finger of the left hand is pushed directly from the right breast to the groin for 21 times.

The ninth formula: the first to the eighth formula shall be completed into 1 degree in sequence, and each time shall be continuously cultivated for 7 degrees. Push and sit on the tray, and press firmly on both knees with both hands. Toes slightly flexed. Rotate the upper body clockwise 21 times. Then, turn it counterclockwise 21 times.

**Quality control**

Test group: After the patient was enrolled in the group, the doctor was responsible for 3 days of training and calibration of the stimulation amount. The mean stimulation amount was (0.5 ± 0.1) kg. Make standard video and operation record book, distribute them to each subject, and require them to fill in the operation record book in time and upload it to the person in charge of quality control. The subjects will use WeChat to take pictures every week, so that the researchers can understand the exercise situation of the participants. The control group: After the patients were enrolled in the group, the psychological counselors conducted a face-to-face psychological evaluation and established a treatment alliance. All cognitive behavior therapy will be assisted by a psychological consultant and a research assistant. The research assistant is responsible for recording the treatment interview content and submitting it to the person in charge of quality control. Subjects will be asked to record daily exercise or learning information according to the treatment content.

**Observations**

**Main indicators**

Multidimensional Fatigue Rating Scale (MFI-20): MFI-20 will be used to assess the degree of fatigue, including 20 items, involving five dimensions: integrity, physiology, spirit, activity and enthusiasm. Likert 5 scoring method is adopted for each item, with 1 point for complete compliance and 5 points for complete non-compliance. Among them, 10 items describing fatigue scored positive, and 10 items describing non fatigue scored negative, with a total score of 20-100. The higher the score, the heavier the fatigue.

**Secondary indicators**

1 Gastrointestinal Symptom Rating Scale (GSRS): GSRS includes 15 items, involving five symptom groups: reflux, abdominal pain, dyspepsia, diarrhea and constipation. Each item is scored by Likert 7, with 1 point for complete compliance and 7 points for complete non-compliance.

2 36 items of the health survey scale (SF-36): there are 36 items and 9 dimensions, including: physiological function, physical pain, general health, vitality, social function, emotional function, mental health and health changes. Each of the nine dimensions is coded and summed separately, and is represented by 0-100. The higher the score, the better the possible health.

3 Nutrition KAP (knowledge, attitude and behavior) questionnaire: the questionnaire is more developed according to previous research. It is composed of three parts, namely, nutrition knowledge, nutrition attitude and nutrition behavior. Each part is coded and summed separately. The total score of the three parts is 100. The higher the score, the better the possible nutritional status of the participants.

4 Food frequency questionnaire: it is divided into two parts: the type of food and the intake frequency of various foods in a certain time. The data of individual food intake in the past period were obtained by calculating the results of the questionnaire. Understand the dietary patterns and habits of the respondents.

5 Bristol Stool Scale (BSFS): BSFS is used to judge the shape of feces. The stool is divided into one of seven types, from type 1 (hard lump) to type 7 (watery diarrhea).

6 Brain gut peptide test: blood will be collected in Yueyang Integrated Traditional Chinese and Western Medicine Hospital. After coagulation, the serum was centrifuged at 3000 rpm for 5 minutes, and stored in a refrigerator at - 80 ℃. Enzyme linked immunosorbent assay (ELISA) was used to detect serum 5-HT, NPY, SP, CGRP levels.

7 Quiescent functional magnetic resonance detection: The 3.0T superconducting magnetic resonance Magnetom skyra imaging system of Yueyang Integrated Traditional and Western Medicine Hospital will be used for functional magnetic resonance data acquisition, which has 32 head specific coils. The scanning parameters of bold functional magnetic resonance imaging are as follows: whole brain, repetition time (TR)=2000 ms, echo time (TE)=30 ms, field of vision (FOV)=250 mm × 250mm, turning angle (FA)=90 °, base=256mm × 256mm, slice thickness/gap=4.0/1mm, voxel size=1.0 ×  1.0   × 1.0, axial slice=33 layers, 240 layers in total. Participants will be asked to take a 10 minute rest before functional magnetic resonance imaging data collection. During data collection, participants will lie on their back and close their eyes. Foam head is used to reduce head movement and earplugs are used to reduce the impact of noise. Participants should stay awake and avoid thinking. The functional magnetic resonance imaging data of participants in both groups will be measured at baseline and 12 weeks after treatment.

8 Detection of intestinal flora: Fresh fecal samples were collected from hospital participants and frozen in - 80 ℃ refrigerator for 3 hours. E. Z.N.A. soil DNA kit (Omega Bio Tek, Norcross, Georgia, USA) will be used for microbial DNA extraction. The V3-V4 hypervariable region of the 16S rRNA gene will be amplified by PCR using 338F (5 '- ACTCCTACGGGGGAGGGAGAG-3') and 806R (5 '- GGACTACHVGGGTWTCTAAT-3'), and then sequenced using Illumina MiSeq PE300. Upload the original data to the NCBI SRA database.

**Efficacy evaluation criteria (determined by MFI-20 score reduction rate)**

Cure: clinical main symptoms and concurrent symptoms completely disappeared, MFI-20 score decreased by ≥ 95%.

Remarkable effect: disappearance of main and concurrent symptoms ≥ 2/3, MFI-20 score reduction ≥ 70%.

Effective: clinical main symptoms and concurrent symptoms disappeared ≥ 1/3, MFI-20 score decreased ≥ 30%.

Ineffective: clinical main symptoms and complications disappeared<1/3, MFI-20 score decreased<30%.

Effective rate=[(score before treatment - score after treatment)/score before treatment] × 100%

Total effective rate=cure rate+apparent efficiency+effective rate

**Statistical analysis**

Input all data into Microsoft Excel to establish a database. SPSS24.0 statistical software was used for data analysis. The measurement data with normal distribution are expressed as "mean ± standard deviation", the measurement data that do not conform to the normal distribution are expressed as "median, interquartile interval", and the counting data are expressed as "frequency, percentage"; If the test of normal distribution and homogeneity of variance is satisfied, the comparison of changes at multiple time points before the study, 6 weeks after the intervention, 12 weeks after the intervention, and 6 weeks of follow-up between the two groups will use ANOVA of repeated measurement design data. If the test of normal distribution and homogeneity of variance is not satisfied, H test will be used. Inspection standard α Is 0.05, when P ＜ 0.05, the difference is statistically significant.
