# Supplementary material for: Effect of Prolong-life-with-nine-turn-method (Yan Nian Jiu Zhuan) Qigong on fatigue and gastrointestinal function in patients with chronic fatigue syndrome: Study protocol for a randomized controlled trial
Source: PLoS One. 2023 Nov 3;18(11):e0287287. doi: 10.1371/journal.pone.0287287 (PMC10624268; doi:10.1371/journal.pone.0287287)
Supplement: S4 File — (PDF) [file pone.0287287.s004.pdf]

Notice on the approval of projects supported by the National Natural Science Foundation of China and related matters

Mr. / Ms. Yao Fei:

In accordance with the regulations of the National Natural Science Foundation of China and the expert review opinions, the National Natural Science Foundation of China (hereinafter referred to as the natural science foundation of China) has decided to approve your application for funding. Project approval No.: 81774443, project name: Research on the regulation mechanism of brain intestinal axis of Yannian jiuzhuan method in the intervention of chronic fatigue syndrome, direct cost: 500000 yuan, project start and end date: January 2018 to December 2021. The review opinions and modification opinions of relevant projects are attached.

Please log in to the NSFC network information system as soon as possible （https://isisn.nsfc.gov.cn）, obtain the project plan supported by the National Natural Science Foundation of China (hereinafter referred to as the plan) and fill in it as required. For the items with modification opinions, please timely adjust the relevant contents of the plan according to the modification opinions; If there is any objection to the modification opinions, it must be put forward before the deadline for submission of the electronic version of the plan. Note: Please fill in the capital budget table of the plan in strict accordance with the measures for the administration of funds for projects funded by the National Natural Science Foundation of China. The amount listed in the subjects of labor fee and expert consulting fee shall not be increased compared with the application.

The electronic version of the plan is through the NSFC network information system （https://isisn.nsfc.gov.cn） Upload it and submit it to NSFC for review after being reviewed by the supporting unit. If the approval fails, return to modify

Submit after; Those who pass the review shall be printed as the paper version of the plan (in duplicate, double-sided printing), which shall be reviewed by the supporting unit and stamped with the official seal of the unit, and then submitted to the project material receiving working group of NSFC. The contents of the electronic version and the paper version of the plan shall be consistent.

National Natural Science Foundation
Department of Medical Sciences
August 17, 2017

Notice on approval of projects supported by the National Natural Science Foundation of China (lump sum project)

Mr. / Ms. Xie Fangfang:

In accordance with the regulations of the National Natural Science Foundation of China, relevant project management measures and expert review opinions, the National Natural Science Foundation of China (hereinafter referred to as NSFC) decides to fund the project you applied for. Project approval No.: 82105038, project name: Study on brain gut interaction mechanism of Yannian jiuzhuan method in CFS based on fMRI brain functional connection density, funding: 300000 yuan, project start and end date: January 2022 to December 2024. The review opinions and modification opinions of relevant projects are attached.

Please log in to the NSFC network information system as soon as possible （ https://isisn.nsfc.gov.cn ）, carefully read the instructions for filling in the project plan supported by the National Natural Science Foundation of China, and fill in the project plan supported by the National Natural Science Foundation of China (hereinafter referred to as the plan) as required. For the items with modification opinions, please timely adjust the relevant contents of the plan according to the modification opinions; If you have any objection to the amendment, you must submit it to the relevant scientific department before the deadline for submission of the electronic version of the plan.

Please send the electronic version of the plan through the NSFC network information system （ https://isisn.nsfc.gov.cn ） Submitted to NSFC after being reviewed by the supporting unit. If the NSFC fails to pass the examination, the returned electronic version of the plan shall be revised before submission; Those who pass the review shall print the paper version of the plan (in duplicate, double-sided printing) and sign in the commitment column of the project leader. The supporting unit shall affix the official seal of the supporting unit on the commitment column, and set the paper signature and seal page of the application in one of the plans, and then submit it to the project material receiving working group of NSFC. The paper version of the plan shall be consistent with the electronic version approved. NSFC will review the paper signature and seal page of the application, and allow the relying unit to modify or supplement the existing problems.

National Natural Science Foundation

October 12, 2021

Ethical review approval

Approval No	2022-077
Entry Name	Study on the characteristics of brain intestinal axis of Yannian jiuzhuan method in the intervention of chronic fatigue syndrome
Sponsor	Fei Yao
Research unit	Shanghai University of traditional Chinese Medicine;<br>Yueyang integrated traditional Chinese and Western Medicine Hospital Affiliated to Shanghai University of traditional Chinese Medicine
Main researcher	Yanbin Cheng
Review category and method	Initial review: meeting review
Review date	January 21, 2022
Review committee	Li Zheng, Xuewen Wang, Lingling Xu, Xiao Shi, Wuquan Sun, Weifeng Liu, Yu Peng, Shouquan Feng, Tingting Zhang, Chunyan Zhang, Minsheng Fan, Li Ren, Yongqi Yao
Review comments	According to the ethical principles of the measures for ethical review of biomedical research involving human beings (2016), nmpa code for the quality management of drug clinical trials, code for the management of clinical trials of medical devices (2016), wma Helsinki Declaration and COMS international ethical guide for human biomedical research, the project is in line with the ethical principles after review by this ethical committee.<br>Please follow GC ρ In principle, clinical research shall be carried out in accordance with the scheme approved by the ethics committee to protect the health of the subjects. Before the research, the applicant shall complete the clinical trial registration.<br>If the main investigator is changed during the study, the applicant shall submit the amendment for review if there is any modification to the clinical research plan, informed consent, recruitment materials, etc. In case of serious adverse events, the applicant shall submit a serious adverse event report in time.<br>Please follow up and review regularly according to the annual review frequency specified by the ethics committee, and the applicant shall submit the research progress report one month before the deadline: the sponsor shall submit the summary report of the research progress of each center to the ethics committee of the team leader unit; In case of any situation that may significantly affect the test or increase the risk of subjects, the applicant is requested to submit a written report to the ethics committee in time.<br>Subjects who do not meet the inclusion criteria or exclusion criteria are included in the study, subjects who comply with the provisions of the suspension of the trial but do not withdraw from the study, giving wrong treatment or dose, giving combination drugs prohibited by the protocol and other situations that do not comply with the protocol to carry out the study, or may have adverse effects on the rights and interests of subjects / health and the scientificity of the study, The sponsor / ombudsman / researcher is requested to submit a violation report.<br>If the applicant suspends or terminates the clinical study in advance, please submit the suspension / termination study report in time to complete the clinical study, and the applicant shall submit the study completion report.
